# Qualitative and quantitative interpretations of the least restrictive means

**DOI:** 10.1111/bioe.12548

**Published:** 2019-01-18

**Authors:** Morten F. Byskov

**Affiliations:** ^1^ Wageningen Universiteit en Researchcentrum Wageningen Netherlands

**Keywords:** capability approach, principle of the least restrictive means, public health ethics, qualitative freedom, quantitative freedom

## Abstract

Within healthcare ethics and public health ethics, it has been the custom that medical and public health interventions should adhere to the *principle of the least restrictive means*. This principle holds that public health measures should interfere with the autonomous freedom of individuals to the least possible or necessary extent. This paper contributes to the discussion on how best to conceptualize what counts as the least restrictive means. I argue that we should adopt a novel, *qualitative* interpretation of what counts as the least restrictive means. Based on the multidimensional framework of the capability approach, the qualitative interpretation holds that the least restrictive means should be measured in terms of whether it restricts *certain normatively valuable freedoms*. I contrast this interpretation with *quantitative* interpretations that measure *how much*, or the extent to which, a public health measure interferes with the freedom of individuals.

## 1 INTRODUCTION

Within healthcare ethics and public health ethics, it has been the custom that medical and public health interventions should adhere to the *principle of the least restrictive means*.^1^ In general, the principle of the least restrictive means holds that public health measures should interfere with the autonomous freedom of individuals to the least possible or necessary extent. As a tool for applying the principle of the least restrictive means in practice, the Nuffield Council of Bioethics has developed the influential ‘intervention ladder’, which visualizes increasingly restrictive public health control measures as rungs on a ladder.^2^ However, it is not always clear what is meant by this principle.

This paper contributes to the ongoing discussion on how best to conceptualize what counts as the least restrictive means. I argue that we should adopt a novel, *qualitative* interpretation of what counts as the least restrictive means. Based on the multidimensional framework of the capability approach, the qualitative interpretation holds that the least restrictive means should be measured in terms of whether it restricts *certain normatively valuable freedoms*. I contrast this interpretation with more common, *quantitative* interpretations that measure *how much*, or the extent to which, a public health measure interferes with the freedom of individuals.

The paper is structured as follows. In Section 2, I provide a short introduction to the concept of the least intrusive means within healthcare ethics ad public health ethics and present the intervention ladder proposed by the Nuffield Council of Bioethics. In Section 3, I argue that the intervention ladder exemplifies a quantitative interpretation of the least restrictive means. In Section 4, I introduce and develop a qualitative interpretation of the principle of the least restrictive means based on the capability approach. In Section 5, I present and discuss three objections to the intervention ladder and the quantitative interpretation that it exemplifies. Finally, in Section 6, I argue that unlike either of the quantitative interpretations, the qualitative, capability‐based interpretation of the least restrictive means can address all of the three objections raised against the intervention ladder.

## 2 THE LEAST RESTRICTIVE MEANS AND THE INTERVENTION LADDER

In order to curb the spread of infectious diseases, public health professionals often implement measures, such as mandatory vaccination, mandatory screenings of at‐risk individuals or the isolation of infected patients. Many of these measures have the potential to be quite intrusive to the freedom of affected individuals, which raises the question of to what extent these measures are justified. Within the literature on public health ethics and healthcare ethics, it has often been argued that public health measures should respect or adhere to the *principle of the least restrictive means*.^3^ According to this principle, public health measures should restrict the freedom of individuals to the least extent possible and/or necessary.^4^ For example, as long as it does not lead to an increased risk of a disease spreading, voluntary vaccination would be preferable to mandatory programmes.

The principle of the least restrictive means was introduced by Childress et al. as an example of especially two (of the five) conditions necessary for the justification of public health interventions, namely *proportionality* and *least infringement*.^5^ The condition of proportionality holds that in the context of a measure that promotes public health yet infringes on some more general moral consideration – such as individual autonomy, privacy, confidentiality or prior government promises – it must be shown that ‘the probable public health benefits outweigh the infringed general moral considerations’.^6^ In other words, it must be shown that the infringement is proportional to the benefit to public health: isolating someone with a common cold for days would not usually be considered a proportionate response because of the low cost of treating the common cold and the low risk it poses to public health. If a public health measure meets the condition of proportionality, and can be shown to be the kind of measure that is both effective and necessary to address the particular public health concern, the least infringement condition then holds that the degree to which a general moral consideration is infringed should be to the lowest degree consistent with achieving the public health goal.

The principle is also sometimes referred to as the principle of the least *intrusive* means or the least restrictive *alternative*. While the terms are usually used synonymously within the literature, the semantic difference might be interpreted to carry different connotations. I here use the term ‘the least restrictive means’, as it refers more specifically to infringements on individual freedom and autonomy. Consider, for example, the distinction made in the claim by Childress et al. that ‘when a policy infringes autonomy, public health agents should seek the least *restrictive* alternative; when it infringes privacy, they should seek the least *intrusive* alternative.’^7^ This focus on freedom and autonomy makes sense, especially, in the context of infectious disease control, where interventions often infringe on people's freedom and autonomous decision making, for example by restricting access to public areas or imposing antibiotic treatments.

The principle of the least restrictive means is often grounded in the normative value of individual autonomy.^8^ For example, the Nuffield Council of Bioethics follows J. S. Mill in arguing that state interventions that infringe on an individual's autonomy can only be justified with the aim to prevent harm to others,^9^ *not* to the individual herself.^10^ As Griffiths and West argue, there are both instrumental and intrinsic justifications of this principle, according to Mill:

Instrumentally, Mill argued that individuals are the best judges of what is in their own interest. But he also suggested that the very purpose of our social institutions is, or should be, the promotion of individual liberty in the sense of reflective self‐determination. So minimizing state interference is intrinsically desirable.^11^

In other words, because, or insofar as, we regard individuals to have the capacity for self‐determination, we ought to protect their freedom to make autonomous choices. Thus, at the core of the principle of the least restrictive means is a commitment to reduce the impact of public health measures on individual freedom to the least extent necessary or possible.

How can the principle of the least restrictive means be put into practice? How can it help us to evaluate actual public health measures? One influential instantiation of the principle of the least restrictive means, and the normative value of autonomy, is the ‘intervention ladder’, proposed by the Nuffield Council of Bioethics.^12^ Table 1 presents an adapted version of the intervention ladder that applies it to the context of infectious disease control.

**Table 1:** The intervention ladder, adapted from the Nuffield Council on Bioethics to the context of infectious disease control

	Name	Description
**1**	Eliminate choice	Regulate in such a way as to entirely eliminate choice, for example through compulsory isolation of patients with infectious disease
**2**	Restrict choice	Regulate in such a way as to restrict the options available to people, for example by limiting access to public spaces during disease outbreaks
**3**	Guide choice through disincentives	Fiscal and other disincentives can be put in place to influence people not to pursue certain activities, for example by fining parents who do not bring their children in for regular check‐ups
**4**	Guide choices through incentives	Regulations can be offered that guide choices by fiscal and other incentives, for example by providing subsidies for screenings and tests
**5**	Guide choices through changing the default policy	Change the default option such that people will have to actively opt out of the preferred choice. For example, instead of waiting for parents to choose whether or not to have their children vaccinated, make vaccines the default option for children
**6**	Enable choice	Enable individuals to change their behaviours, for example by offering regular health consultations
**7**	Provide information	Inform and educate the public, for example as part of public campaigns to promote good hygiene habits
**8**	Do nothing or simply monitor the situation	–

The ladder should be read in the following way, according to the authors. At the bottom of the ladder, we find the least restrictive alternative, namely to do nothing or simply monitor the situation. This is the least restrictive means because it does not involve any restrictions on the autonomous choice of the individual. Moving up the ladder, the higher rungs aim to increasingly influence the choice of the individual by providing additional information, guidance, incentives and disincentives. At the top of the ladder, we find the most restrictive means, which involve, first, a decrease in alternative options and, finally, the complete elimination of choice.

The higher up the ladder one goes, it is argued, the stronger the justification of the implemented means needs to be. The authors clearly hold that a stronger justification is necessary because of the increased infringement on individual freedom and autonomy, the higher up the ladder one goes. Consider, for example the following statements: ‘The most intrusive is to legislate in such a way as to *restrict the freedom* significantly’; ‘A more intrusive policy initiative is likely to be publicly acceptable only if it is clear that it will produce the desired effect *and this can be weighed against the loss of liberty that will result*’; and, ‘the benefits to individuals and society should be weighed against *the erosion of individual freedom*’.^13^

It is possible, however, to restrict the freedom of individuals in different ways and it is not exactly clear in what way the authors intend the intervention ladder to be interpreted. In particular, it is possible to advance both quantitative and qualitative interpretations of what it means to restrict freedom to the least extent possible. On the *quantitative interpretation,* the least restrictive means is the measure that restricts the range or amount of freedoms that an individual can choose from to the least extent possible, while the *qualitative interpretation* holds that the least restrictive means should be measured in terms of whether it restricts certain normatively valuable freedoms.

The difference between these two interpretations is evident in how they can be applied in practice to evaluate and trade‐off competing public health measures. As argued at the top of the section, within public health it is often necessary to make difficult decisions that balance effective intervention with a concern for protecting individual autonomous freedom, and that interventions should adhere to the least restrictive means in this regard. Yet, how we conceptualize individual freedom – that is, in terms of quantity or quality – determines how we balance or trade‐off these concerns.

Consider, for example, the obesity epidemic and the alternative measures that might be taken to address it. WHO estimates that there are more than 200 million adults and 18 million children under 5 years, who suffer from obesity worldwide. In non‐industrialized countries, more than 115 million individuals are estimated to suffer from ‘obesity‐related problems’, including diabetes, cardiovascular diseases, hypertension, risk of stroke and some forms of cancer. While the most efficient and effective ways of addressing obesity would arguably include taxing junk food or even banning sugary drinks in public institutions, proponents of the quantitative interpretation would likely object to such measures because they restrict individual freedom. Taxes on junk food, they could argue, are more likely to be a burden on less affluent individuals, who consume more of it and have fewer resources to purchase more healthy alternatives, while banning sugary drinks constitutes a direct infringement on individuals’ freedom of choice. Thus, according to the proponents of the quantitative interpretation of the least restrictive means, it would be preferable to implement measures that are less restrictive and more conducive to individual responsible choice, such as public‐awareness campaigns, even if they are less effective to address the obesity problem.

Proponents of the qualitative interpretation of the least restrictive means would not be so quick to dismiss the more restrictive measures. Essentially, whether or not such wider‐ranging interventions are permitted on the qualitative interpretation depends on whether they violate any normatively relevant or valuable freedoms. As I argue in Section 4, what counts as normatively relevant or valuable might be highly context‐specific, but in the case of the alternative measures proposed above, proponents of the qualitative interpretation are likely to arrive at different conclusions than proponents of the quantitative interpretation. For example, while they might agree that a tax on junk food is too restrictive out of a normative concern for equality, they might be less inclined to object to the ban on sugary drinks in public institutions because, *ex hypothesi*, buying sugary drinks does not constitute a valuable choice in and of itself. Moreover, and contrary to the preferred option on the quantitative interpretation, insofar as a healthy lifestyle is arguably a valuable choice and insofar as public‐awareness campaigns are less effective at promoting healthy lifestyles, proponents of the qualitative interpretation might object to such interventions because they risk interfering with individuals’ actual freedom to achieve a healthy lifestyle, for example by not addressing personal obstacles, such as *akrasia*, or structural issues, such as socioeconomic inequalities, which are important drivers of obesity.

This raises the question that I will address in the remainder of this paper: when push comes to shove, should public health professionals prioritize the concern for the amount of alternative choices that people have (i.e., the quantitative interpretation) or the concern for whether the choices that people have represent valuable options (i.e., the qualitative interpretation) when choosing between alternative public health measures?

In the following two sections I introduce and discuss these two interpretations in greater detail and show how the intervention ladder exemplifies the quantitative interpretation. In Section 5, I argue that the overlap with the quantitative interpretation makes the intervention ladder vulnerable to three objections that, as I argue in Section 6, only can be addressed by adopting the qualitative interpretation of the least restrictive means.

## 3 THE QUANTITATIVE INTERPRETATION OF THE LEAST RESTRICTIVE MEANS

The first interpretation of the principle of the least restrictive means focuses on *how much*, or the extent to which, a public health measure interferes with the range of freedoms and choices that individuals have. I call this the *quantitative interpretation* of the least restrictive means because it is primarily concerned with the quantity of alternative options that people have and the extent to which public health measures interfere with and restrict this range of options.

The quantitative interpretation of the least restrictive means builds on the (libertarian) claim that more freedom is better than less freedom. According to this view, freedom is something that be measured by ‘counting’ the range of alternative options that an individual can choose from.^14^ The ability to choose between different options has value in itself, on this view, regardless of what choices these options represent, because it enhances individual autonomy.^15^ As Carter argues, if autonomy is defined by making one's own choices in life, it stands to reason that having more options to choose from is more conducive to the exercise of autonomy than having fewer options.^16^ Thus, someone is better off if she can choose between three options, *x, y* and *z*, rather than merely two options, *x* and *y*, and the least restrictive means is accordingly the one that protects the widest range of choices or, conversely, restricts the least amount of alternative options that an individual can choose from.

If we take autonomy to be the primary moral concern, in this way, the quantitative interpretation is intuitively attractive because it aims to leave as much opportunity for autonomous choice as possible. Consider, for example, two public health measures that constrain the social interactions of an individual carrier of an infectious disease in order to prevent contamination. Both measures allow that the carrier can be with his family but bar him from certain public spaces. However, while the first measure only restricts our carrier from going to work, the second measure additionally prevents him from visiting his friends. If we are concerned with the individual carrier's autonomy, we should, on the quantitative interpretation, say that the first measure is the least restrictive measure since it allows him greater freedom to exercise his autonomy: whereas the first measure allows our carrier to choose between two options of social interaction (family, friends), the second measure only allows one, namely being with his family.

I have claimed that the intervention ladder (table 1), which I briefly introduced in the previous section, exemplifies quantitative interpretation of the principle of least restrictive means. How is this? Ignoring the bottom rung, which does not aim to make any restrictions, the intervention ladder applies the quantitative interpretation of the least restrictive means in two analytically distinct, but, in practice, overlapping ways.

First, the lower rungs on the intervention ladder (rungs 3–7) work by increasing *the degree to which* the means aim to guide individuals to choose a preferred option, without directly enforcing this choice. For example, as argued above, by providing incentives and disincentives, it is possible to influence the choice of individuals without taking away alternative options. Consider, for example, a public health measure that works through disincentives by fining individuals that do not report for regular health check‐ups. While this measure does not restrict the option (and thus the autonomy) of individuals in a direct sense – they are still free to choose whether or not to report for check‐ups – the disincentive does aim to make the option of not attending check‐ups less attractive and thereby influence the choice, even if the individual would have preferred *not* to attend regular check‐ups. Because the individual choice thereby is directed away from what the individual *would have* chosen had the risk of being fined not been present, we should say that such external influence is antagonistic to truly autonomous choice: significant financial pressure effectively amounts to the elimination of certain options for people with limited economic means and hence these interventions can be considered as *indirect* restrictions on individual freedom.

The second way in which the intervention ladder exemplifies the quantitative interpretation of the least restrictive means occurs on rungs 2 and 1 – restrict choice and eliminate choice, respectively – where the ladder *directly* decreases *the amount of* choices that individuals have. For example, we might restrict access to public spaces during outbreaks in order to curb further contamination. While this still leaves other options open for individuals to choose from, it removes at least one option from the set of choices that individuals have. The most restrictive means on the intervention ladder, according to the quantitative interpretation, is the enforcement of a particular choice, such as mandatory vaccines, because it leaves no alternative options to be chosen.

These two ways can work (a) separately, (b) simultaneously, or (c) in conjunction. In the first case, (a) a public health measure aims to promote a particular choice either by providing incentives and disincentives or by decreasing the alternative options individuals can choose from. In the second case, (b) a public health measure promotes a particular choice both by providing incentives and disincentives and by decreasing alternative options at the same time. For example, in an attempt to contain or prevent an outbreak, we may, at the same time, enforce mandatory immunization *and* encourage safe social behaviours, such as staying away from public spaces when exhibiting symptoms.

Finally, (c) it is possible to implement a public health measure that restricts individual freedom *both* directly and indirectly. For example, enforcing mandatory vaccinations arguably interferes with an individual's choice both directly and indirectly. Such a measure is an infringement both in terms of the degree to which it influences choice and in terms of the reduction of alternative options. Likewise, the pressure might be so strong that the individual has no real choice. Less affluent individuals, for example, might find that they have no choice but to report for check‐ups if the alternative is a costly fine.

It might be objected that the authors of the intervention ladder did not have this quantitative interpretation in mind when setting it out. When applying the intervention ladder in practice, it might be argued, it is necessary to, and in practice public health professionals do, invoke other normative values, such as equality and justice, when deciding on alternative measures.

However, the ladder still suggests that, *prima facie*, having more choice is better than having less. Moreover, the quantitative interpretation is a consistent philosophical view of freedom and an interpretation of the least restrictive means that is promoted by recent libertarian and neo‐liberal approaches to public health and healthcare. On this view, for example, public health interventions that are seen as paternalist, such as mandatory vaccinations or smoking bans, are considered to be problematic because they reduce individual opportunities for autonomous choice, including the individual's responsibility for her own health.^17^

With a structure that equates no interference (rung 8) with the least restrictive means and identifies full paternalistic intervention (rung 1) as the most restrictive means, it is clear how the intervention ladder *can* be interpreted in this quantitative, libertarian and anti‐paternalist way. Hence, the question is whether the least restrictive means – and the intervention ladder – *should* be interpreted in such quantitative terms. As we shall see in Section 5, the quantitative approach to determining the least restrictive alternative is vulnerable to at least three objections that can only be addressed by adopting a qualitative interpretation of the least restrictive means. In the next section, I proceed to introduce this qualitative interpretation.

## 4 A QUALITATIVE, CAPABILITY‐BASED INTERPRETATION OF THE LEAST RESTRICTIVE MEANS

The least restrictive means in quantitative terms is not always the measure that is the most desirable, neither from a public health perspective nor from the point of view of individual agents. In the first case, as argued, less restrictive means might be less effective to achieve public health aims than interventions that interfere more with the choices of individual agents. In the second case, the least restrictive means in quantitative terms might fail to protect certain qualitative concerns, such as socioeconomic equality.

Contrary to the quantitative interpretation, the second way in which the principle of the least restrictive means can be interpreted aims to take these qualitative considerations of freedom into account when deciding on public health measures. Accordingly, I label this the *qualitative interpretation* of the least restrictive means. The qualitative interpretation determines the least restrictive means based on whether certain normatively valuable capabilities are being restricted, protected or even expanded.

The capability approach has, in recent decades, gained prominence as an alternative theory of well‐being.^18^ At the core of the capability approach is a normative commitment to conceptualize well‐being in terms of *capabilities* and *functionings*. Capabilities are defined as the real freedom that people have to do certain things or be a certain kind of person; functionings are capabilities that have been realized. Examples of these *doings* and *beings* are travelling, falling in love, getting an education, voting, being healthy, being politically active and being married. The notion that capabilities are *real freedoms* means that they are opportunities that are cleared of all possible obstacles such that someone can turn them into achieved functionings if she should so wish. Someone may, for example, have the capability to travel in the sense that no one restricts her, she has the money to buy a plane ticket and access to an airport. Yet, at the same time she may not have the corresponding functioning of travelling if she has not yet undertaken her trip.

Crucially, the capability approach takes a multidimensional and pluralistic view of freedom and well‐being.^19^ An individual can be affected by a particular public health measure in different ways.^20^ For example, the isolation of infected patients may not only affect their freedom to move around, but also their ability to engage in social relations and participate in recreational activities. Likewise, two different individuals may experience different impacts from the same public health measure. Someone who is less socially active would be less affected by being isolated from friends and family than someone who values social relations higher.

By focusing on what people are able to do or be (i.e., their capabilities), the capability approach aims to broaden the informational basis of traditional behavioural models and evaluative accounts.^21^ What is missing from these models, according to Sen, is an account of how people value the different choices that they have and what makes certain freedoms more valuable or normatively important than others. As Sen argues, a singular concern with the quantity of freedoms that individuals have would be unsustainable because it would:

… be then possible to assess the freedom of a person independently of – or prior to – the assessment of alternatives between which the person can choose … It is odd to conclude that the freedom of a person is no less when she has to choose between three alternatives which she sees respectively as ‘bad’, ‘awful’, and ‘gruesome’ than when she has the choice between three alternatives which she assesses as ‘good’, ‘excellent’, and ‘superb’.^22^

Thus, Sen concludes, ‘[t]he assessment of the elements in a range of choice has to be linked to the evaluation of the freedom to choose among that range.’ That is, what matters is not only or primarily the range of choices that one has, but rather whether the alternative options are regarded as valuable choices.

How can the capability approach be applied to the interpretation of the principle of the least restrictive means? On the capability approach, the focus is on the promotion and protection of *certain normatively relevant* doings and beings. That is, most capability scholars hold that some freedoms are more valuable or normatively relevant than others.^23^ Interpreting the principle of the least restrictive means through the capability perspective means that we are primarily concerned with protecting those capabilities that we consider to be normatively relevant or valuable, which may sometimes lead to trade‐offs with the commitment to protect public health.^24^

What capabilities should be protected by the principle of the least restrictive means, according to the qualitative interpretation? The qualitative interpretation should be supplemented with a thick description or theory of valuable capabilities and functionings that can tell us what restrictions of individual freedom and autonomy are normatively problematic. While some capability scholars have proposed a universal list of capabilities that is owed to all individuals, other scholars have argued that normatively relevant and valuable capabilities must be specified to the particular circumstances.^25^ Both approaches can be useful for specifying what capabilities are normatively relevant to the principle of the least restrictive means.

Objectively, there are certain capabilities that we consider to be more relevant for well‐being than others.^26^ For example, it is more important to have access to (adequate) healthcare than it is to be able to choose between two equally effective brands of washing liquid. These capabilities might indeed be quite basic. In the context of infectious disease control, there are often good reasons to restrict an infected individual's freedom of movement or right to engage with social relations. Hence, a universal list of basic capabilities that no public health measure can violate would arguably be limited to those that are necessary for human survival and living a decent human life, such as access to healthcare, proper nutrition and adequate living accommodation.

More contextually, different individuals might value different freedoms higher than others, relative to their needs, socioeconomic position and/or sociocultural norms and values. For example, a disabled individual who relies on the informal care of family members would likely be more affected by being subject to isolation than someone who is able to care for herself. Hence, on the qualitative interpretation, isolating her from her relations, or failing to provide alternative care arrangements, would constitute a greater restriction for the disabled individual than it would for the able‐bodied person.

How does this differ from the quantitative interpretation? Whereas the quantitative interpretation counts any restriction of the range of individual freedoms – whether directly or indirectly – as a negative, the capabilitarian interpretation holds that only some restrictions are normatively problematic, namely those that unduly and excessively restrict people's normatively valuable capabilities. Hence, this interpretation is qualitative, rather than quantitative, in the sense that it is compatible with certain restrictions *as long as these do not interfere (excessively) with people's protected capabilities*.

## 5 THREE OBJECTIONS TO THE QUANTITATIVE INTERPRETATION

I have in the previous two sections presented two alternative interpretations of the least restrictive means – one quantitative in nature; the other qualitative. I have argued that the Nuffield Council of Bioethics’ intervention ladder at present is best interpreted as an example of the quantitative interpretation. The question is, however, whether the quantitative interpretation of the least restrictive means – and, by extension, the intervention ladder – should be preferred when deciding between public health interventions.

A general problem with adopting a quantitative interpretation of the intervention ladder, according to its critics, is that it thereby adheres to a single value, namely individual freedom or liberty. The bottom rungs of the ladder represent the least restriction of personal freedom, while the upper rung represents full coercion. As Griffiths and West argue, ‘[o]n this conception, any intervention designed to promote public health necessarily comes at a cost to individual autonomy.’^27^

This singular commitment to personal freedom is subject to at least three objections. The quantitative interpretation of the intervention ladder ignores (a) that we are also concerned with other values than individual freedom when evaluating and implementing public health measures, (b) that some interventions are necessary to protect and expand individual freedom, and (c) that some freedoms are normatively more relevant, important or valuable than others. In the following I argue that the quantitative interpretation (and, hence, the intervention ladder) is subject to these three objections and show how they can be addressed by adopting the qualitative interpretation of the least restrictive means.

### 5.1 Concern for other values than freedom

First, as Dawson argues, we are also concerned with other values than individual freedom when evaluating and implementing public health measures, such as whether they are effective at minimizing the spread of an infectious disease.^28^ Yet, the least restrictive means, measured in terms of individual freedom, might not be at all effective in this regard. Although it constitutes the least restrictive means, according to the intervention ladder, doing nothing would be irresponsible during a disease outbreak. Arguably, if we were concerned with public health and controlling the spread of the disease, we would need an even stronger justification of doing nothing than to actually implement a measure that to some extent restricts the freedom of infected individuals. For this reason, Dawson argues, we need to embrace value pluralism when evaluating and implementing public health measures:

Expanding or contracting a single value, such as liberty, is almost certainly going to interact with and impact on other values … We may need to decide how much liberty we are willing to sacrifice to bring about greater equity or greater well‐being.^29^

Dawson's objection can be avoided if we consider the principle of least restrictive means to include a *ceteris paribus* clause, however. According to such a clause, the least restrictive means is the one that restricts individual freedom to the least extent possible *after* having taken into account other (normative) concerns and values, such as the effectiveness, feasibility, fairness, well‐being and equality. If two measures are equally effective and respectful of people's well‐being, we should prefer the measure that is least restrictive of individual freedom. What Dawson's objection does highlight, though, is that identifying the least restrictive means is not as straightforward as merely being concerned with individual freedom since, all things considered, other concerns and values also matter.

The qualitative, capability‐based interpretation, by comparison, can address the first objection in two ways. First, as Robeyns and Qizilbash argue, the capability approach is an open‐ended and underspecified framework that can be further specified into more particular capability theories.^30^ While the capability approach at its core is normatively committed to conceptualize people's well‐being in terms of their real freedom to do or be certain things, when developing a particular capability theory – such as a capabilitarian interpretation of the least restrictive means – it is necessary to take other normative concerns into consideration, such as equality, equity and efficiency. In other words, neither the capability approach in general nor its more specific capability theories are exclusively concerned with individual freedom.

Secondly, the capability approach acknowledges that people are not only concerned with their individual freedom, but also other values, such as fairness, equality, equity and efficiency. What this means for the interpretation of the least restrictive means is that it cannot simply be reduced to the measure that restricts individual freedom to the least extent, measured in quantitative terms as a range of options or choices. Rather, if we care about these additional values, the least restrictive means would be the one that is most cost efficient in terms of balancing individual freedom and effectiveness or the one that preserves the most equal amount of freedom among affected individuals.

### 5.2 Some freedom‐restricting interventions are necessary to protect valuable freedoms

The second objection points out that not all interventions restrict the range of options that individuals are free to choose from.^31^ For example, as on rungs 6 and 7, the enabling of choice, by providing additional options, and the provision of information seem actually to enhance the freedom of the individuals. Offering access to eradication treatment is not antithetical to individual freedom of choice.^32^ In fact, it provides infected individuals with a greater freedom to combat their disease – an opportunity that they might not have, or only would have had to a lesser extent, had this access not been provided.

It might be argued that the above example only shows that the relevant kind of intervention is one that also entails some interference with autonomous choice. However, moving up the ladder, providing incentives (rung 4) constitutes an indirect interference with individual choices, as argued in the previous section, yet may lead to a gain in terms of individual freedom overall. For example, if the cost of vaccinations has hitherto made it impossible for lower‐income individuals to become vaccinated, subsidizing voluntary vaccinations would actually enable this choice for these individuals.

Proponents of the quantitative version of the intervention ladder could respond to this in two ways. First, they could argue that they are primarily or merely concerned with protecting negative freedom (i.e., the freedom *from* being interfered with) but that the above example shows a gain in positive freedom (i.e., the freedom *to* vaccinate). The subsidy provided still constitutes a more restrictive means since it aims to interfere with the autonomous choice of individuals. Secondly, it is doubtful whether interventions higher up the ladder – providing disincentives and the removal of choices – enhance individual (positive) freedom in this way. For example, providing a disincentive, such as a fine, does not, as we saw in the previous section, enhance the freedom of less affluent individuals to vaccinate – quite the opposite, in fact.

We can likewise respond to these amendments in two ways. First of all, it is wholly implausible to reduce individual freedom to only concern the negative aspects freedom, ignoring the gain in positive freedom. For example, for someone to have the negative freedom to choose between vaccinating or not (i.e., no one will interfere with this choice), they would also need to have the positive freedom to vaccinate. Since, in the above example*,* the positive freedom to vaccinate only became available to the lower‐income individuals qua the proposed subsidy, their negative freedom from being interfered with when choosing whether or not to vaccinate only became available as a result of the subsidy. It would be absurd to claim that the lower‐income individuals in the example were free, negatively speaking, to vaccinate as long as no one would interfere with this choice if the option to vaccinate did not, positively speaking, appear in their range of options to choose from. Secondly, the point here is not to show how *all* public health measures lead to more freedom, but rather to show that *some* interferences with autonomous choices actually lead to more freedom.

Thus, the quantitative interpretation fails to accommodate the fact that some interventions actually enhance individual freedom, measured in terms of the range of options that one can choose from, because, on this interpretation, any kind of interference, whether indirectly by way of incentive or disincentive or more directly by taking away alternative options, potentially counts as a restriction of individual freedom.

By comparison, the qualitative, capability‐based interpretation acknowledges that some interventions may be necessary in order to ensure individual freedom. This is not only in regards to the observation of social contract theory that in order to ensure the equal freedom of everyone, some restrictions on individual freedom must be accepted. This, as Dawson notes, seems to already be explicit in the Nuffield Council of Bioethics report, though not in the intervention ladder.^33^

Rather, as Sen has argued, individual freedom cannot be reduced merely to non‐interference.^34^ It is possible for someone to enjoy non‐interference in exercising a certain choice, yet lack the actual freedom to achieve that choice. Consider, for example, a public health measure that works by enabling an additional choice (rung 6 on the intervention ladder), such as bimonthly health‐checks at the local clinic. While this measure might provide an additional choice for able‐bodied individuals, it is of little value to disabled individuals who suffer from reduced mobility. Unless the measure provides (compensation) for transportation to and from the clinic or in‐home consultations, such a measure does not actually enable a choice for disabled individuals.

### 5.3 Some freedoms are normatively more relevant, important or valuable than others

The third objection holds that a quantitative interpretation of the intervention ladder fails to accommodate the idea that some freedoms are normatively more relevant, important or valuable than others. For example, the freedom to care for one's children is more valuable than the freedom to choose between two equally effective brands of washing liquid.^35^ If this is true, then interventions that interfere with fewer, yet more valuable freedoms might actually be more objectionable than interventions that interfere with more, yet less valuable freedoms.

Consider, for example, how parents can be assumed to care for the health and well‐being of their children.^36^ Yet, many parents are not always able to make healthiest choices for their children for several reasons: because they cannot afford healthy lifestyle choices, because they are unaware of what a healthy lifestyle entails, and/or because they simply lack the willpower to consistently choose healthy lifestyle choices. Moreover, these issues are compounded by societal influences, such as how grocery stores will often place candy in a way that appeals to children and at prices that render the economic cost of making unhealthy choices relatively small. If parents do value the health of their children yet for various reasons lack the capability to make healthier lifestyle choices, they should (and arguably would) also value the implementation of health interventions that restrict or seek to curb unhealthy choices, such as increased health education, mandatory gym classes, taxes or bans on candy in grocery stores, and regulations on how candy can be marketed and, for example, the size of sugary drinks.

The quantitative interpretation of the least restrictive means fails to address the third objection because it cannot meaningfully distinguish between the restrictions of valuable freedoms. In the above example, in a society where the health of children is valued higher than making unhealthy choices, interventions to curb child obesity would be more acceptable than leaving the option for unhealthy choices open, even if they constitute more restrictive interventions on the quantitative interpretation. If the primary concern is merely individual freedom, any measure that involves either a loss in alternative choices or involves a (dis)incentive would constitute a (more) restrictive means. From that perspective being able to choose between equally efficient brands of washing liquid is just as important as being able to care for one's children.

The qualitative interpretation, as argued, clearly holds that some freedoms are normatively more valuable than others and that the principle of the least restrictive means, therefore, should aim, primarily, to protect those valuable freedoms. On the one hand, there may be some capabilities that we find objectively and fundamentally valuable for human lives and well‐being, such as the access to healthcare; on the other hand, the restriction of certain capabilities might be more problematic for some individuals than others, such as the access to care for disabled individuals. These normatively important aspects can only be taken into account if the principle of the least restrictive means is supplemented with a thick, qualitative description or theory of valuable capabilities and functionings.

In sum, the three objections discussed in this section point to important aspects that need to be taken into account when setting out public health measures and which the quantitative interpretation, as exemplified by the intervention ladder, cannot satisfy. Hence, any alternative interpretation of the principle of the least restrictive means should be able to address these three objections. I have argued that the qualitative, capability‐based interpretation of the least restrictive means can meet this challenge.

## 6 ADDRESSING TWO OBJECTIONS TO THE QUALITATIVE INTERPRETATION

Even if the qualitative interpretation can address these three objections, it is possible to raise two additional objections to it. First, it might be objected that, by focusing on certain normatively valuable freedoms, the qualitative interpretation ignores the quantitative aspect of freedom and autonomy. That is, this objection correctly argues, the extent to which a public health measure leaves room for autonomous choice – both in terms of range of choices as well as in terms of non‐interference – *also* matters for how we should evaluate it. If this is true, the focus of the qualitative interpretation, the objection concludes, is misguided or at least incomplete.

While the observation that the amount of autonomous choice one has matters for the evaluation of public health measures is indeed true, it fails as an objection to the qualitative interpretation. The reason for this is that that the qualitative interpretation *does not* hold that the quantity of freedom is irrelevant, only that the least restrictive means cannot be *reduced* to the measure that protects the highest amount of choices or, conversely, restricts the least amount of choices. What the notions of capabilities and functionings highlight, is that in order for someone to have the *real* (or what is sometimes called *substantive*) freedom to do or be something, it is necessary also to provide certain positive conditions. In other words, in order properly to conceptualize the least restrictive means, it is insufficient merely to look at how much or how little public health measures interfere with the freedom of individuals. We also need to look at the extent to which these measures (fail to) provide the positive conditions necessary for the exercise of this freedom and whether these freedoms are normatively relevant or valuable.

Second, proponents of the quantitative interpretation might object that they are not merely concerned with the amount of freedom that people have. That is, they might argue, non‐quantitative judgments are inescapable when conducting normative analyses about the relative weight to be given to different public health measures. Recall, for example, the case of obesity in Section 2 and how proponents of the quantitative interpretation might take concerns for equality (i.e., taxes on junk food unequally affect the choices of less affluent individuals) into consideration when deciding on appropriate measures to address obesity.

Although this example indeed shows how the quantitative interpretation of the least restrictive means can take qualitative concerns into account, this concern can still be reduced to a quantitative concern, namely a concern that the amount of freedom of some individuals is reduced, rather than a concern with the actual qualitative notion of social and economic equality. An actual concern for socioeconomic equality might lead public health professionals to accept a loss in individual freedom of choice, but proponents of the qualitative interpretation are not ready to make this sacrifice. Thus, they face a dilemma. On the one hand, they can insist, as I argued in Section 5, that the quantitative interpretation includes a *ceteris paribus*‐clause, holding that only after taking all things into consideration should we choose the measure that is quantitatively superior, in which case the quantitative interpretation is actually *not* a quantitative position because it gives priority and precedence to qualitative concerns. On the other hand, avoiding this reduction into a qualitative interpretation, the proponents of the quantitative interpretation could reaffirm the commitment to prioritize the concern for individual freedom of choice by insisting that the least restrictive means is the one that protects the largest amount of individual options or, conversely, restricts the least amount of alternative choices.

Accordingly, I contend that we should adopt a qualitative interpretation of the least restrictive means. While both interpretations are concerned with the value of freedom, the qualitative interpretation holds that the infringement of public health measures on individual autonomy should first and foremost be measured, not in terms of the amount of overall freedom that they restrict, but rather in terms of whether certain valuable freedoms are infringed upon. Taking into account the quality of the freedoms that individuals have to choose from better reflects the values that are at stake when implementing public health measures, including the general moral considerations that Childress et al. highlight, and might even be more reflective of actual public health practice in which public health professionals and decision makers *do* put such non‐quantitative concerns front and centre.^37^ Moreover, it provides a rebuke to the libertarian strand of public health ethics and policy.

Lastly, it should be noted that I have not here investigated whether this qualitative, capability‐based interpretation is compatible with the intervention ladder or whether it entails that we should abandon the ladder as an illustration of the least restrictive means. However, as Dawson concludes, ‘if we count more than liberty as relevant, we cannot use the intervention ladder’.^38^ I will let it be subject to further research whether this holds true and, if so, how to best represent a qualitative, capability‐based interpretation of the least restrictive means.

## 7 CONCLUDING REMARKS

This paper contributes to the literature on the principle of the least restrictive means in at least three ways. First, I argue that there are not one but two analytically distinct interpretations of the principle, namely quantitative and qualitative interpretations. Second, I discuss and further develop two objections that have been raised against the most influential instantiation of the principle of the least restrictive means, namely the Nuffield Council of Bioethics’ intervention ladder, and add a novel, third objection. Third, I present a qualitative, capability‐based interpretation of the principle of the least restrictive means. According to the qualitative interpretation, the least restrictive means is not necessarily the measure that interferes the least with individual freedom, but rather the measure that protects certain normatively valuable capabilities. I have argued that this qualitative interpretation fares better against the three objections raised to the quantitative interpretation of the Nuffield Council of Bioethics’ intervention ladder and that it can accommodate the aspect of quantitative freedom. For these reasons, I contend that we should adopt the qualitative, rather than a quantitative, interpretation of the principle of the least restrictive means.

## CONFLICT OF INTEREST

The author declares no conflict of interest.

## NOTES

1 Childress, J. F., Faden, R. R., Gaare, R. D., Gostin, L. O., Kahn, J., Bonnie, R. J., … Nieburg, P. (2002). Public health ethics: Mapping the terrain. *Journal of Law, Medicine & Ethics*,* 30*(2), 170–178.

2 Nuffield Council on Bioethics. (2007). *Public health: Ethical issues*. London, UK: Nuffield Council on Bioethics.

3 Holm, S. (1995). Not just autonomy – The principles of American biomedical ethics. *Journal of Medical Ethics*,* 21*(6), 332–338; Beauchamp, T. L., & Childress, J. F. (2013). *Principles of biomedical ethics*. New York, NY: Oxford University Press; Childress et al., *op. cit*. note 1; Gillon, R. (2003). Ethics needs principles – four can encompass the rest – and respect for autonomy should be ‘first among equals’. *Journal of Medical Ethics*,* 29*(5), 307–312; Nuffield Council on Bioethics, *op. cit*. note 2; Buchanan, D. R. (2008). Autonomy, paternalism, and justice: Ethical priorities in public health. *American Journal of Public Health*,* 98*(1), 15–21; Eyal, N. (2014). Nudging by shaming, shaming by nudging. *International Journal of Health Policy & Management*, 3(2), 53–56; Saghai, Y. (2014). Radically questioning the principle of the least restrictive alternative: A reply to Nir Eyal, comment on ‘Nudging by Shaming, Shaming by Nudging’. *International Journal of Health Policy & Management*,* 3*(6), 349–350; Allen, T., & Selgelid, M. J. (2017). Necessity and least infringement conditions in public health ethics. *Medicine, Health Care and Philosophy*, 1–11.

4 Hence, the principle of the least restrictive means is often accompanied by the condition that the means should still be effective enough to achieve its intended aim. For example, Gostin argues that ‘[t]he standard does not require officials to use less‐than‐optimal interventions’. Allen and Selgelid, on the contrary, argue that this conditional is redundant since it is implied by the principle that the public health measure in question should also be necessary and/or effective for achieving the intended public health goal. See Upshur, R. E. G. (2002). Principles for the justification of public health intervention. *Canadian Journal of Public Health/Revue Canadienne de Sante'e Publique*,* 93*(2), 101–03; Gostin, L. O. (2005). Public health preparedness and ethical values in pandemic influenza. In S. L. Knobler, A. Mack, A. Mahmoud, & S. M. Lemon (Eds.), *The threat of pandemic influenza: Are we ready? Workshop summary*. Washington, DC: National Academies Press; Dawson, A. (2016). Snakes and ladders: State interventions and the place of liberty in public health policy. *Journal of Medical Ethics*,* 42*(8), 510–513; Allen & Selgelid, *op. cit*. note 3.

5 Childress et al., *op. cit*. note 1.

6 Ibid: 173.

7 Ibid: 173 (emphasis added).

8 Childress et al., *op. cit*. note 1; Buchanan, *op. cit*. note 3; Dawson, A., & Verweij. M. (2008). The steward of the millian state. *Public Health Ethics*,* 1*(3), 193–195; Dawson, *op. cit*. note 4.

9 Within the context of infectious disease control, it is not exactly clear whether we should define the actions of one individual to be harmful to other individuals in physical or more intangible terms. Moreover, as Verweij argues, within infectious disease control, it may be more relevant to focus on whether a harmful action was the result of negligent, rather than intentional, behaviour. Krom, A. (2011). The harm principle as a mid‐level principle? Three problems from the context of infectious disease control. *Bioethics*,* 25*(8), 437–444; Verweij, M. (2011). Infectious disease control. In A. Dawson (Ed.), *Public health ethics: Key concepts and issues in policy and practice* (pp. 100–117). Cambridge, UK: Cambridge University Press.

10 Nuffield Council on Bioethics, *op. cit*. note 2; Mill, J. S. (1991). On liberty. In J. Gray (Ed.), *On liberty and other essays* (pp. 5–128). Oxford, UK: Oxford University Press.

11 Griffiths, P. E., & West, C. (2015). A balanced intervention ladder: promoting autonomy through public health action. *Public Health*,* 129*(8), 1092–1098.

12 Nuffield Council on Bioethics, *op. cit*. note 2.

13 Ibid: 41–42.

14 Carter, I. (1999). *A measure of freedom*. Oxford, UK: Oxford University Press; Van Hees, M. (2010). The specific value of freedom. *Social Choice and Welfare*,* 35*(4), 687–703; Van Hees, M., & Wissenburg, M. (1999). Freedom and opportunity. *Political Studies*,* 47*(1), 67–82.

15 Carter, I. (1995). The independent value of freedom. *Ethics*,* 105*(4), 819–845.

16 Carter, I. (2004). Choice, freedom, and freedom of choice. *Social Choice and Welfare*,* 22*(1), 73.

17 For examples and discussions of this view, see Buchanan, *op. cit*. note 3; Fahlquist, J. N. (2006). Responsibility ascriptions and public health problems. *Journal of Public Health*,* 14*(1), 15–19; Nys, T. (2008). Paternalism in public health care. *Public Health Ethics*,* 1*(1), 64; Wiley, L. F., Berman, M. L., & Blanke, D. (2013). Who's your nanny? Choice, paternalism and public health in the age of personal responsibility. *Journal of Law & Medical Ethics*,* 41*(suppl), 88–91.

18 Robeyns, I. (2016). Capabilitarianism. *Journal of Human Development and Capabilities*,* 17*(3), 397–414; Robeyns, I. (2016). The capability approach. In E. N. Zalta (Ed.), *The Stanford encyclopedia of philosophy*. Stanford, CA: Metaphysics Research Lab, Stanford University.

19 Alexander, J. M. (2004). Capabilities, human rights and moral pluralism. *The International Journal of Human Rights*,* 8*(4), 451–469; Nussbaum, M. (2003). Capabilities as fundamental entitlements: Sen and social justice. *Feminist Economics*,* 9*(2–3), 33–59; Sen, A. (2005). Human rights and capabilities. *Journal of Human Development*,* 6*(2), 151–166.

20 Entwistle, V. A., & Watt, I. S. (2013). A capabilities approach to person‐centered care: Response to open peer commentaries on ‘Treating patients as persons: A capabilities approach to support delivery of person‐centered care’. *The American Journal of Bioethics*,* 13*(8), W1–W4.

21 Sen, A. (1974). Informational bases of alternative welfare approaches: Aggregation and income distribution. *Journal of Public Economics*,* 3*(4), 387–403; Sen, A. (1979). Issues in the measurement of poverty. *Scandinavian Journal of Economics*,* 81*(2), 285–307; Sen, A. (1979). Personal utilities and public judgements: Or what's wrong with welfare economics. *The Economic Journal*,* 89*(355), 537–558; Sen, A. (1979). Utilitarianism and welfarism. *Journal of Philosophy*,* 76*(9), 463–489.

22 Sen, A. (1993). Capability and well‐being. In M. Nussbaum & A. Sen (Eds.), *The quality of life* (pp. 30–53). Oxford, UK: Clarendon Press.

23 Ibid.

24 See also Saghai for a similar conclusion: ‘Because the least restrictive alternative is not always the easiest or least costly to implement, the requirement does not and should not apply when the state interferes with nonfundamental liberties.’ Saghai, *op. cit*. note 3, p. 350.

25 Claassen, R. (2011). Making capability lists: Philosophy versus democracy. *Political Studies*,* 59*(3), 491–508; Byskov, M. F. (2018). Methods for the selection of capabilities and functionings. In F. Comim, S. Fennell, & P. B. Anand (Eds.), *New frontiers of the capability approach*. Cambridge, UK: Cambridge University Press.

26 Nussbaum, M. (2011). *Creating capabilities. The human development approach*. Cambridge, MA: Belknap Press of Harvard University Press; Claassen, R., & Düwell, M. (2013). The foundations of capability theory: Comparing Nussbaum and Gewirth. *Ethical Theory and Moral Practice*,* 16*(3), 493–510.

27 Griffiths & West, *op. cit*. note 11, p. 1093.

28 Dawson, *op. cit*. note 4.

29 Ibid: 513.

30 Robeyns, *op. cit*. note 18; Qizilbash, M. (2010). Interpreting the capability approach: Thick and thin views. *Human Rights Defender*, 5–7; Qizilbash, M. (2012). The capability approach: Its interpretation and ‘limitations’. In F. Panzironi & K. Gelber (Eds.), *The capability approach. Development practice and public policy in the Asia–Pacific region* (pp. 9–22). London/New York: Routledge.

31 Griffiths & West, *op. cit*. note 11, p. 1093.

32 Dawson, *op. cit*. note 4.

33 Ibid.

34 Sen, A. (1988). Freedom of choice: Concept and content. *European Economic Review*,* 32*(2–3), 269–294.

35 Williams, B. (1987). The standard of living: interests and capabilities. In G. Hawthorn (Ed.), *The standard of living* (pp. 94–102). Cambridge, UK; Cambridge University Press.

36 Promoting the health and well‐being of our children can be seen as both subjectively and objectively valuable. Subjectively, it is something that parents *do* arguably value; objectively, the health of children is valuable because it is an intrinsic part of their well‐being as humans.

37 Childress, *op. cit*. note 1.

38 Dawson, *op. cit*. note 4, p. 513.

